# Effects of combined physical education and nutritional programs on schoolchildren’s healthy habits

**DOI:** 10.7717/peerj.1880

**Published:** 2016-04-11

**Authors:** Maria Chiara Gallotta, Sara Iazzoni, Gian Pietro Emerenziani, Marco Meucci, Silvia Migliaccio, Laura Guidetti, Carlo Baldari

**Affiliations:** 1 Department of Movement, Human and Health Sciences, University of Rome “Foro Italico,”Rome, Italy; 2 Department of Health and Exercise Science, Appalachian State University, Boone, NC, United States

**Keywords:** Children, Nutritional intervention, MVPA, Physical education, School context

## Abstract

**Background.** A multidisciplinary approach seems to be effective in creating healthy habits in children. The aim of this study was to evaluate the efficacy of three 5-month combined physical education (PE) and nutritional interventions on body composition, physical activity (PA) level, sedentary time and eating habits of schoolchildren.

**Methods.** Anthropometric data, weekly PA level, sedentary time and eating habits of 230 healthy students were analysed using a repeated-measures ANOVA with Group (experimental group 1 vs experimental group 2 vs control group), Adiposity Status (under fat vs normal fat vs obese), and Time (pre vs post) as factors.

**Results.** Body fat mass percentage increased after intervention (18.92 ± 8.61% vs 19.40 ± 8.51%) in all groups. The weekly PA level significantly increased after intervention in both experimental groups. Sedentary time significantly decreased after the intervention period (565.70 ± 252.93 vs 492.10 ± 230.97 min/week, *p* < 0.0001). Moreover, obese children were more sedentary than under fat and normal fat children. Children significantly changed the consumption of some specific foods after intervention.

**Discussion.** This study revealed the effectiveness of a combined PE and nutritional intervention to improve children’s healthful dietary practices and to encourage an active lifestyle. However, it needs a further appropriate development to establish patterns of healthful dietary practices that encourage an active lifestyle with which to maintain healthy habits through life.

## Introduction

Obesity is a complex clinical condition with a multifactorial origin linked to the obesogenic environment we live in Chaput, Doucet & Tremblay (2012). Low levels of physical activity (PA), together with sedentary behavior and poor diet are preventable causes of childhood obesity, besides possible genetic determination (EPIGCHRCA, 2011).

Youth overweight incidence is stabilizing in several countries, with approximately 10% of children and adolescents in overweight condition and 2–3% in obesity condition (Cole & Lobstein, 2012). Several regions and countries have particularly high rates of pediatric obesity: more than 30% of children and adolescents in the Americas, while approximately 20% in Europe are overweight or obese. More than 30% of Italian children are overweight or obese (Nardone et al., 2015; Spinelli et al., 2015). The survey conducted by the Italian Surveillance system *OKkio alla Salute* reported that levels of physical inactivity and sedentary behaviors in Italian children are high (Spinelli et al., 2011). Specifically, the survey revealed that only 18% of children were engaged in structured PA or sport for more than 1 h a week, while about 35% of children spent their time in sedentary activities (television viewing and playing video-games) for more than 2 h a day (Spinelli et al., 2011; http://www.epicentro.iss.it/okkioallasalute). Moreover, the same survey highlighted the prevalence of inappropriate eating habits such as skipping breakfast, consuming abundant mid-morning snack and sugary drinks, consuming irregularly fruit and/or vegetables which can lead to weight gain among children (Spinelli et al., 2011; http://www.epicentro.iss.it/okkioallasalute). Specifically, 8% of children skipped breakfast; 31% of children ate unbalanced breakfast in terms of carbohydrates and proteins; 52% ate a mid-morning snack too abundant; moreover, 22% of parents declared that their children consumed irregularly daily fruits and/or vegetables. Finally, 44% of children habitually consumed sweet drinks (Spinelli et al., 2011; http://www.epicentro.iss.it/okkioallasalute). Interestingly, regular practice of PA and relative physical fitness level of children and adolescents have declined in the past few decades (Tremblay et al., 2010). Low levels of PA in childhood, tracking into adulthood, combined with a non-correct diet (specially, fruit and vegetables low consumption) are associated with adverse health outcomes, including greater adiposity, increased cardio-metabolic risk factors, poorer bone mineralization, behavioral problems, low self-esteem, and also, poorer academic attainment (Kipping et al., 2014). Therefore, overweight and obesity prevention should require a combined intervention in order to control all modifiable factors which contribute to childhood and adolescence obesity. Educational interventions to obtain changes in life habits during childhood could be a key strategy, since it is more difficult to establish healthy habits during adulthood rather than during childhood (Llargues et al., 2011). A recent study highlighted the importance of planning integrated and multi sectorial actions in school-based programmes to promote correct dietary and motor habits and for the control of body weight (Sacchetti et al., 2015). In this perspective, school might be a crucial setting to obtain changes in obesity related behavior and to induce positive effects on eating habits, PA level and sedentary behaviors in children (http://www.a-g-a.de; Kavey et al., 2003). The school environment seems to be the ideal setting for the promotion of PA, providing to a great number of children and adolescents opportunities to be physically active during physical education (PE) classes (Wechsler et al., 2000; Verrotti et al., 2014). School also provides a valid possibility to train in an appropriate manner. Moreover, since children usually consume one/two meals per day in school (Khambalia et al., 2012), it could promote appropriate consumption of food. Although studies focused on a single intervention strategy, involving PA or dietary component or reducing sedentary behaviors, showed positive effects on adiposity outcomes (Amini et al., 2015), multidisciplinary interventions seem to be more successful in preventing, managing, and treating pediatric obesity (Amini et al., 2015) and, importantly, its comorbidities (Bryan et al., 2011), encouraging children to establish a long-lasting healthy habits. However, systematic reviews of combined school-based PA and nutritional interventions reported controversial results about the efficacy of comprehensive intervention including diet and exercise to manage and prevent children’s obesity (Brown & Summerbell, 2009; Kelishadi & Azizi-Soleiman, 2014; Amini et al., 2015). Plachta-Danielzik et al. (2011) showed that one-year health-promotion intervention had positive effects on BMI but not on lifestyle habits of primary school children. Fairclough et al. (2013) proposed the project “CHANGE!”, a school-based PA and healthy eating intervention of 20 weeks, which induced positive changes on body size of 10–11 years-old children, and no effects on PA level, sedentary time and food intake. Contrarily, Rausch Herscovici, Kovalskys & De Gregorio (2013) reported that a six-month obesity prevention program positively improved dietary intake and food consumption of 9–11 years-old children but did not affect their anthropometric measures. Finally, Bacardí-Gascon, Pérez-Morales & Jiménez-Cruz (2012) reported favourable effects of a six-month intervention on lifestyle (food consumption and PA) and obesity of 2nd and 3rd grade elementary school children.

Therefore, the aim of the present study was to evaluate the effectiveness of different PE programs, in combination with a nutritional intervention, on schoolchildren’s healthy habits. We compared body composition, PA level, sedentary time and eating habits of primary school children before (pre) and after (post) three different five-month PE interventions in combination with the same nutritional intervention. These PE interventions were as follows: experimental intervention 1, conducted by a specialist PE teacher and structured according to the ministerial programs of PE for primary school (Decree of the President of the Republic of Italy of 16 November 2012, 2013); experimental intervention 2, conducted by a specialist PE teacher and focused on highly varied activities aimed to contribute to a multilateral development of coordinative abilities (Roth, 1982); and finally control intervention, conducted by the generalist teacher and structured according to the ministerial programs of PE for primary school (Decree of the President of the Republic of Italy of 16 November 2012, 2013). We hypothesized that a multidisciplinary intervention could allow children to establish healthy habits, positively affecting their PA and dietary patterns. In this perspective, quality PE could act as the groundwork for a lifelong engagement in PA and sport. The learning experiences offered to children through PE lessons could help them to acquire psychomotor skills, cognitive understanding, social and emotional skills that they need to lead a physically active life. In spite of this overwhelming evidence, only 60% of Italian primary schools offer at least two hours per week of PE (http://www.epicentro.iss.it/okkioallasalute). The PE lessons are conducted during curricular time by generalist teachers who do not have specific expertise in PE teaching. For this reason, we proposed professionally guided PE interventions to allow children to participate in well-structured PA programs conducted by specialist PE teachers. We also hypothesized that experimental intervention 2 could be more effective in inducing children’s active lifestyle. The specialist PE teacher, articulating enjoyable, developmentally appropriate and varied experiences of PA, provided children a variety of opportunities that could meet their personal interests and individual differences (Lewis, 2014). Therefore, the school practice of new and unusual activities that provide more choice of activities could encourage PA participation (Lewis, 2014) in out-of-school settings, reducing sedentary behaviors.

## Material & Methods

### Study design and setting

The study was designed as a cluster-randomized controlled intervention in all classes (from Grade 3 to Grade 5) of three primary schools in the rural area in the north of the city of Rome (Italy). The unit of randomization, intervention, and analysis is the participating school (Thomas, Nelson & Silverman, 2015). Sixteen classes with a total of 230 children between 8 and 11 years of age volunteered to participate in this study.

### Selection of schools and allocation to intervention

The study focused on schools located in a rural area about 50 km north of the city of Rome (Italy). The area was included in a 5 km radius circle from a central reference point in order to obtain a sample with comparable environmental characteristics. This comprised a total of five schools. Eligibility criterion for the schools was the participation to the European School Fruit Scheme. This EU-wide voluntary scheme provided school children with fruit and vegetables, aiming to encourage good eating habits in young people (European Commission Agriculture and Rural Development: School Fruit Scheme; http://ec.europa.eu/agriculture/sfs/). We further excluded those schools that were already engaged in PA programmes/interventions. The remaining three schools were invited and agreed to participate in the study. The schools were the experimental units that received the intervention and therefore were the units that were either randomized to intervention or control group (Thomas, Nelson & Silverman, 2015).

### Participants

The classroom demographics broke down to 88 Grade 3 children (8–9 years of age), 72 Grade 4 children (9–10 years of age) and 70 Grade 5 children (10–11 years of age). After cluster randomization, 78 participants (33 girls and 45 boys) were in the experimental group 1 (ExpG1), 83 participants (38 girls and 45 boys) in the experimental group 2 (ExpG2), and the remaining 69 (29 girls and 40 boys) participants in the control group (CG).

Children were also classified into three groups: under fat (UF), normal fat (NF) or overweight/obese (OB) children based to their body fat mass percentage (FM%) according to the McCarthy’s age-sex specific cut-offs (McCarthy et al., 2006). Specifically, children were subdivided into UF: under fat (FM% < 2nd centile); NF: normal fat (2nd centile < FM% < 85th centile); OB: overweight/obese (FM% > 85th centile). Therefore, 21 UF, 36 NF and 21 OB children were in the experimental 1 group, 23 UF, 40 NF and 20 OB children were in the experimental 2 group, 29 UF, 28 NF, 12 OB children were in the control group.

The University Ethical Committee approved this investigation (Rif 3502 Prot. 1883/15). Written informed consent and assent was obtained from both parents and children prior to study participation.

### Anthropometric measurements

Pre- and post-intervention anthropometric measurements assessed children’s weight, height, BMI *z*-score, lean body mass and body fat mass percentage (FM%). Weight and height were measured using a scale and a stadiometer to the nearest 0.5 kg and 0.1 cm, respectively. BMI *z*-score was calculated for each BMI measure with reference to age-and sex- specific limits (Cole et al., 2000; Cole et al., 2005). Moreover, for each child, FM% and lean body mass (kg) were measured by multi-frequency hand-to-foot bioelectrical impedance method (IOI 353 analyzer; Jawon Medical Co. Ltd, Seoul, South Korea).

### Physical activity measurement

Pre- and post-intervention PA level was assessed by the Italian version of the Physical Activity Questionnaire for Older Children (PAQ-C) (Gobbi, Ferri & Carraro, 2012). It is a self-administered, 7-day recall instrument. The questionnaire consists of nine questions about sports and games, physical activities at school, and those during leisure time, including the weekend. Each question is scored from 1 to 5, with the final score obtained through the means of the question scores (Crocker et al., 1997).

### Sedentary time measurement

Pre- and post-intervention self-reported sedentary time was assessed by parental proxy interview. Children’s parents were asked to report the average number of minutes spent reading, television viewing, playing video-games, using computer per week day and weekend day, outside of school time (Coombs et al., 2013).

### Eating habits measurement

Eating habits of children were assessed pre- and post-intervention by a seven day diet record (Vereecken et al., 2008). All children were asked how many times weekly they usually ate pasta and rice, bread, potatoes, legumes, vegetables, meat, fish, eggs, cold cuts, fruit, sweets, drinks, milk, dairy product and snack. The response options for the items were scored as 1 = “*never*,” 2 = “*less than once a week*,” 3 = “*once a week*,” 4 = “*2 to 4 days a week*,” 5 = “*5 to 6 days a week*,” 6 = “*once a day, every day*,” and 7 = “*every day, more than once*.” The test–retest stability correlations of the food items range 0.40–0.83 (Vereecken et al., 2008).

Before administration, children were instructed to fill all questionnaires, that were administered to all children in the classroom, in a quiet condition. Children had adequate time to complete the questionnaires. A researcher was present in the classroom to answer to any children’s questions.

### Healthy nutrition intervention

The nutritional intervention lasted 5 months and was designed to increase consumption of fruit and vegetables, by improving nutritional knowledge. It was based on the nutritional program “European School Fruit Scheme” of the European Commission of Agriculture and Rural Development (http://ec.europa.eu/agriculture/sfs/). The program was launched in the 2009/10 school year (Watson, 2008). The intervention consisted of topics like fruit and vegetables characteristics, nutritional values, biodiversity, seasonality, and territoriality. The topics were taught monthly through methodologies that are linked to children’s system of learning (short lectures/talks, games and sensory workshops). Topics, such as, health/nutrition (eat well to stay healthy), science (I know what I eat), affective and sensory (use and enjoy my senses), environmental (contact with nature and conservation of ecosystems and biodiversity), social (knowledge of territorial identity) were also covered. All topics were taught by the generalist teachers who had previously attended a training course by specialists (e.g., nutritionist, dietician) from the CRA—NUT (Council for Research and Experimentation in Agriculture—Research Center of the Foods and Nutrition) (http://www.fruttanellescuole.gov.it/). In addition, a properly trained staff provided one free piece of fresh fruit (or vegetable) to children each school week, as a snack at mid-morning or mid-afternoon break to increase their consumption of fruit and vegetables in order to establish healthy eating habits. Every child consumed fruits or vegetables at least 36 times during the program, and at least ten different kinds of fruit or vegetable. Finally, an information campaign targeted at parents was conducted by producing and distributing informative material and by creating a specific web site section (http://www.fruttanellescuole.gov.it/contenuti/frutta-casa-0) to prolong the effect of encouraging consumption.

### Physical education intervention

The intervention period lasted 5 months. Experimental interventions differed in type and mode of physical activities in which children were engaged but they were equivalent in structure, overall duration and intensity, and consisted of two 1 h sessions per week. The exercise intensity of both programs was monitored using an OMNI scale (Utter et al., 2002) to avoid possible differences in intensity between the two types interventions. Rice et al. (2015) reported validity coefficients of 0.67–0.87 between OMNI RPE and physiological responses in four age groups of children (6–8, 9–10, 11–12 and 13–15 years of age).

They were designed by the same specialist PE teacher who conducted one of the two weekly lessons; the other was conducted by the generalist teacher supervised by the specialist PE teacher. Each lesson of both interventions included 15 min of warm-up, 35 min of moderate-to-vigorous physical activities (MVPA) within a range of 5 < RPE < 8 (US Department of Health and Human Services, 2008), and 10 min of cooldown and stretching.

OMNI RPE measures were obtained during each MVPA session. Children verbally indicated the number after looking at the scale in order to have an indication of how hard the exertion felt during the exercise session.

The *experimental intervention 1* was designed to promote health, fitness, sensory-motor, social and communicative development. It was primarily focused on endurance, strength, flexibility exercises and circuit training for cardiovascular health (Decree of the President of the Republic of Italy of 16 November 2012, 2013). The specialist PE teacher proposed exercises without high coordinative demands with the main goal of developing children’s physical characteristics (Gallotta et al., 2015), (see Appendix for full protocol description).

The *experimental intervention 2* was focused on improving the coordination and dexterity of the participants. It provided complex movements involving multiple degrees of freedom and interactions between different body parts or with different objects (Egan, Verheul & Savelsbergh, 2007) to develop psychomotor competence and expertise in movement-based problem solving considering various tasks that involved decision-making behaviours. Thus, accurate timing, temporal estimations, temporal production, and spatial adjustments were essential parts of the experimental requirements (Buscà et al., 2011). Moreover, it provided children the opportunity to experience and to learn various sports and unusual activities that are often gender biased. It was organized in four different didactic modules lasting 5 weeks each one. Each module focuses on specific coordination abilities found in sports games, rhythmic activities, gymnastics and/or fitness activities. These activities were previously published by our lab (Gallotta, 2014; Gallotta et al., 2014; Gallotta et al., 2015). The capacities requested were: static and dynamic balance, combination, kinesthetic differentiation, spatial orientation, rhythm and reaction.

The *sport-games module* allowed children to recognize and manage the characteristics of traditional sports games and/or pre-sports (e.g., handball, mini-volleyball, mini-basketball). The educational proposals focused on specific aspects of games: rules, roles, spaces, times and strategies.

The rhythmic activities module (cursive) was planned to specifically develop rhythmic and time perception abilities. Exercises or movement sequences were proposed, with or without tools, using wide execution variability in relation to the perception of some concepts such as “before,” “after,” “contemporary,” “next,” “slow,” “fast” and “cadence.” Sounds and/or music tracks were used.

The *gymnastics module* was characterized by a general movement development. Children were able to become aware of their movement patterns. Therefore, activities were proposed to allow children to be able to manage and vary the movement patterns as a function of spatial and temporal parameters, in executive situations of growing complexity.

The *fitness activities module* was planned to develop children’s strength, endurance, speed and flexibility.

The intensity of experimental intervention 2 gradually increased in terms of complexity and degree of difficulty (concerning movement time, precision, amplitude, size of the target). The intervention was based on the systematic variation of practice by making task demands progressively more difficult (see Appendix for full protocol description).

The *control group* continued to follow the traditional PE school curriculum given by the generalist teacher using traditional PE programmes (Decree of the President of the Republic of Italy of 16 November 2012, 2013).

### Statistical analysis

All results were expressed as mean ± SD. Anthropometric data, weekly PA level, sedentary time data and eating habits data were analysed using a 3 × 3 × 2 mixed-model repeated-measures analysis of variance (ANOVA) with Group (experimental group 1 vs experimental group 2 vs control group), Adiposity Status (under fat vs normal fat vs obese), and Time (pre vs post) as factors. Effect size was calculated using Cohen’s definition of small, medium, and large effect size (as partial *η*^2^ = 0.01, 0.06, 0.14). Significant interactions were further analyzed by means of Bonferroni *post hoc* analysis. Within the Group factor, differences in the baseline anthropometric, weekly PA level, sedentary time and eating habits data were analysed using an ANOVA comparison test.

Statistical significance was conventionally considered as *p* ≤ 0.05.

## Results

All children (100% of the total sample) had pre- and post-intervention measures.

### Anthropometric data

The anthropometric data of children participating the study are reported in Table 1.

**Table 1:** Pre- and post-intervention anthropometric characteristics of under fat (UF), normal fat (NF) or overweight/obese (OB) children in experimental 1 (ExpG1), experimental 2 (ExpG2) and control (CG) group.

	**UF**		**NF**		**OB**	
	Pre	Post	Pre	Post	Pre	Post
**ExpG1**						
Weight (kg)	30.10 ± 4.48	31.01 ± 4.63	36.01 ± 7.65	36.41 ± 7.53	51.20 ± 10.12	52.22 ± 10.12
Height (cm)	133.81 ± 5.63	134.76 ± 5.77	136.66 ± 9.25	137.93 ± 9.05	141.43 ± 8.68	142.86 ± 8.85
BMI *z*-score	−0.14 ± 0.92	0.13 ± 1.01	0.77 ± 0.73	0.68 ± 0.69	2.01 ± 0.32	1.96 ± 0.31
FM (%)	9.04 ± 2.79	10.10 ± 4.56	19.98 ± 3.56	19.66 ± 3.85	29.96 ± 3.78	30.01 ± 3.83
Lean body mass (kg)	27.58 ± 3.79	27.78 ± 3.82	28.60 ± 6.13	29.02 ± 5.96	35.68 ± 6.21	36.36 ± 6.10
**ExpG2**						
Weight (kg)	29.25 ± 3.86	30.01 ± 4.00	36.66 ± 6.32	37.67 ± 6.72	48.63 ± 5.27	49.94 ± 5.59
Height (cm)	135.58 ± 6.85	135.84 ± 7.36	138.56 ± 7.46	138.87 ± 7.73	139.10 ± 7.06	140.15 ± 7.32
BMI *z*-score	−0.7 ± 1.01	−0.58 ± 0.84	0.63 ± 0.78	0.72 ± 0.65	1.99 ± 0.27	1.97 ± 0.30
FM (%)	8.40 ± 3.09	9.02 ± 2.75	19.92 ± 4.22	20.70 ± 4.22	29.78 ± 3.51	30.21 ± 3.54
Lean body mass (kg)	28.15 ± 4.11	27.55 ± 3.72	29.01 ± 7.70	29.53 ± 4.98	34.09 ± 4.01	34.77 ± 4.22
**CG**						
Weight (kg)	31.83 ± 5.03	32.94 ± 5.11	34.34 ± 8.55	35.39 ± 8.89	57.18 ± 7.49	58.76 ± 7.80
Height (cm)	138.20 ± 7.56	139.44 ± 7.69	136.75 ± 9.03	137.62 ± 9.25	144.45 ± 6.28	145.54 ± 6.62
BMI *z*-score	−0.17 ± 0.75	−0.12 ± 0.76	0.33 ± 0.97	0.43 ± 0.94	2.13 ± 0.39	2.10 ± 0.40
FM (%)	8.31 ± 3.29	9.14 ± 2.89	18.80 ± 2.52	19.47 ± 3.06	30.39 ± 3.87	30.74 ± 4.41
Lean body mass (kg)	29.73 ± 4.58	30.63 ± 4.65	29.19 ± 6.53	29.85 ± 6.62	39.60 ± 3.61	40.44 ± 3.98

The main effect of Time revealed that children’s FM% (*F*_1,206_ = 17.28, *p* < 0.0001, *η*^2^ = 0.077) (18.92 ± 8.61% vs 19.40 ± 8.51%) significantly increased after intervention. Body weight (*F*_1,218_ = 150.91, *p* < 0.0001, *η*^2^ = 0.409) (37.69 ± 10.60 kg vs 38.59 ± 10.84 kg), body height (*F*_1,219_ = 92.67, *p* < 0.0001, *η*^2^ = 0.297) (137.89 ± 8.07 cm *vs* 138.84 ± 8.23cm) and lean body mass (*F*_1,189_ = 96.11, *p* < 0.0001, *η*^2^ = 0.337) (30.54 ± 6.10 kg vs 31.12 ± 6.21 kg) also increased after intervention.

### Physical activity data

**Figure 1:**
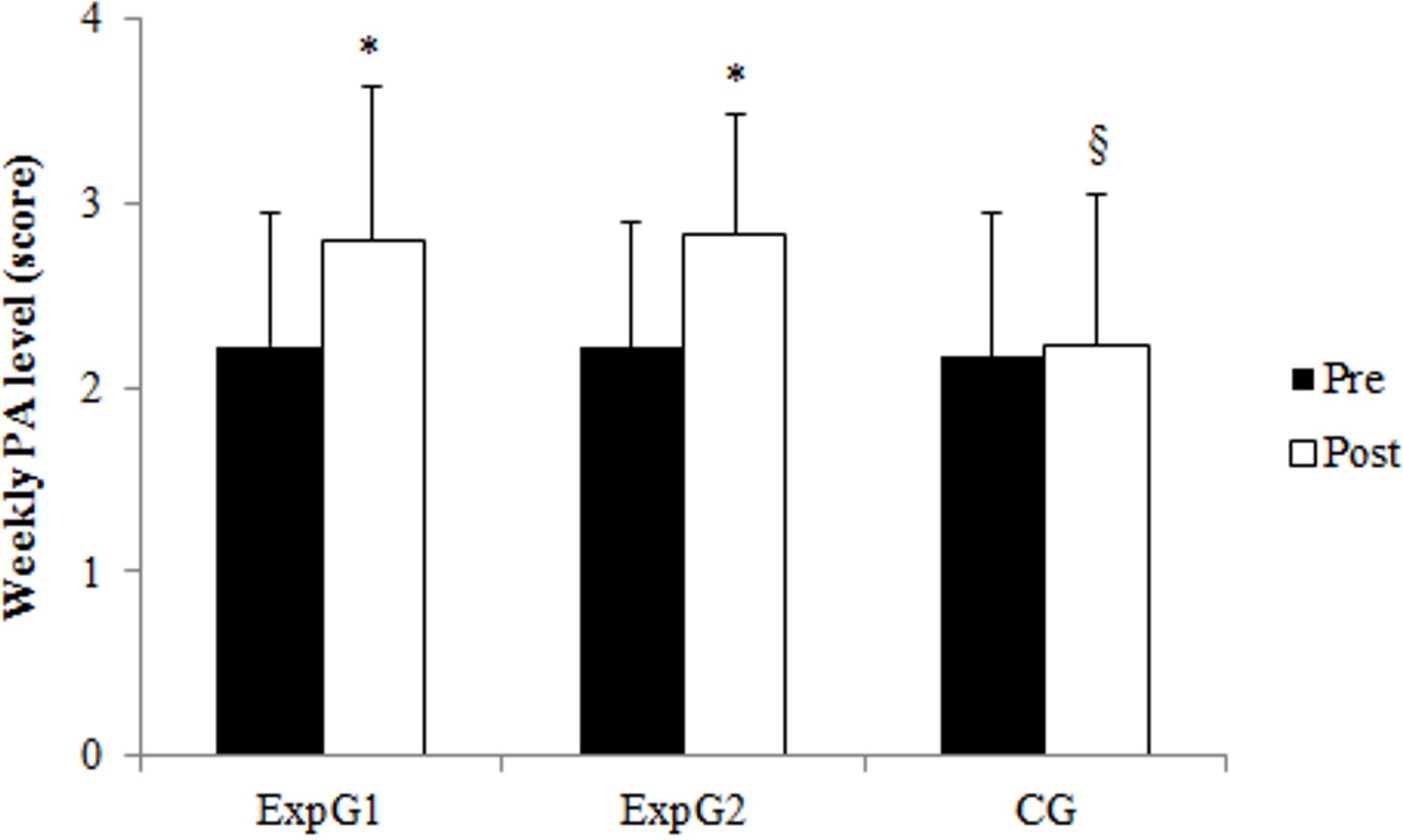
Children’s weekly PA level pre- and post-intervention in experimental 1 (ExpG1), experimental 2 (ExpG2) and control (CG) group. ^∗^*p* < 0.0001 Post vs Pre § *p* < 0.0001 CG vs ExpG1 vs ExpG2.

The main effect of Time (*F*_1,220_ = 122.77, *p* < 0.0001, *η*^2^ = 0.36) revealed that weekly PA level significantly increased after intervention. The main effect of Group (*F*_2,220_ = 5.89, *p* = 0.003, *η*^2^ = 0.05) revealed that control group had lower PA level than experimental 1 and experimental 2 groups (2.2 ± 0.8 score vs 2.5 ± 0.8 score *vs* 2.5 ± 0.7 score, respectively). Moreover, Time × Group interaction (*F*_2,220_ = 22.02, *p* < 0.0001, *η*^2^ = 0.17) revealed that only experimental groups increased PA level after intervention (Fig. 1). Finally, the main effect of Adiposity Status (*F*_2,220_ = 11.08, *p* < 0.0001, *η*^2^ = 0.09) revealed that UF children were more active than NF and OB children (2.7 ± 0.8 score vs 2.3 ± 0.8 score *vs* 2.2 ± 0.8 score, respectively).

### Sedentary time data

The main effect of Time (*F*_1,221_ = 18.85, *p* < 0.001, *η*^2^ = 0.08) revealed that sedentary time significantly decreased in children of all groups (565.70 ± 252.93 vs 492.10 ± 230.97 min/week, *p* < 0.0001). However, the main effect of Adiposity Status (*F*_2,221_ = 36.31, *p* < 0.0001, *η*^2^ = 0.25) revealed that OB children were more sedentary than UF and NF children (703.49 ± 246.75 min/week vs 458.82 ± 228.27 min/week *vs* 492.71 ± 210.86 min/week, respectively).

### Eating habits data

Table 2 reports only significant results relevant for the present study: main effects of Time, Time × Group and Time ×Adiposity Status interactions.

**Table 2:** Significant ANOVA results of children’s eating habits.

Food	Factors	*F*	df	*p*	Partial *η*^2^
Bread	Time × Adiposity Status	3.85	2	0.029	0.03
Potatoes	Time	4.89	1	0.028	0.02
	Time × Group	7.64	2	0.001	0.06
Vegetables	Time	6.37	1	0.001	0.28
Meat	Time	27.33	1	0.001	0.11
Fish	Time × Group	5.18	2	0.006	0.04
Eggs	Time	11.87	1	0.001	0.05
	Time × Group	10.52	2	0.000	0.09
Cold cuts	Time	42.56	1	0.000	0.16
	Time × Group	7.50	2	0.000	0.06
Fruit	Time	15.33	1	0.000	0.06
	Time × Group	4.30	2	0.015	0.04
Sweet	Time	65.25	1	0.000	0.23
	Time × Group	23.40	2	0.000	0.18
Sweet drink	Time × Group	14.48	2	0.000	0.17
Dairy product	Time	4.59	1	0.033	0.02
Snack	Time	45.81	1	0.000	0.17
	Time × Group	11.29	2	0.000	0.09
	Time × Group × Adiposity Status	4.85	4	0.001	0.08

The main effect of Time revealed that children significantly changed the consumption of some specific foods after the intervention period. Specifically, consumption of potatoes (4.52 ± 1.72 time/week vs 4.25 ± 1.81 time/week, *p* < 0.05), meat (3.83 ± 1.38 time/week vs 3.21 ± 1.35 time/week, *p* < 0.05), eggs (3.07 ± 1.28 time/week vs 2.69 ± 1.15 time/week, *p* < 0.05), cold cuts (3.45 ± 1.51 time/week vs 2.65 ± 1.39 time/week, *p* < 0.05), sweets (3.74 ± 1.58 time/week vs 2.89 ± 1.62 time/week, *p* < 0.05), dairy products (3.97 ± 1.56 time/week vs 3.62 ± 1.61 time/week, *p* < 0.05) and snacks (3.29 ± 1.68 time/week vs 2.43 ± 1.40 time/week, *p* < 0.05) decreased after the intervention period. Consumption of vegetables (3.97 ± 1.63 time/week vs 4.29 ± 1.55 time/week, *p* < 0.05) and fruits (4.30 ± 1.64 time/week vs 4.85 ± 1.71 time/week, *p* < 0.05) increased after intervention period.

Moreover, Time × Group interaction indicated the likely presence of differential effects of Group on changes of some foods consumption after intervention (Table 3).

**Table 3:** Children’s food consumption pre- and post-intervention in experimental 1 (ExpG1), experimental 2 (ExpG2) and control (CG) group.

Food	ExpG1		ExpG2		CG	
	Pre	Post	Pre	Post	Pre	Post
Potatoes	3.65 ± 1.49	2.82 ± 1.5^*^	3.49 ± 1.52	3.22 ± 1.26	3.23 ± 1.17	3.65 ± 1.21^*^
Fish	3.14 ± 1.28	2.55 ± 1.20^*^	3.07 ± 1.19	3.23 ± 1.56	3.27 ± 1.27	3.42 ± 1.04
Eggs	2.88 ± 1.13	2.20 ± 1.03^*^	3.28 ± 1.49	2.60 ± 1.03^*^	3.04 ± 1.13	3.36 ± 1.12^*^
Cold cuts	3.21 ± 1.35	2.10 ± 1.19^*^	3.36 ± 1.69	2.46 ± 1.45^*^	3.50 ± 1.45	3.50 ± 1.11
Sweets	4.17 ± 1.61	2.77 ± 1.53^*^	3.22 ± 1.50	2.20 ± 1.39^*^	3.90 ± 1.49	3.87 ± 1.52
Drinks	4.16 ± 1.60	2.76 ± 1.52^*^	3.21 ± 1.49	1.49 ± 1.38^*^	3.94 ± 1.57	3.56 ± 1.44
Snacks	3.42 ± 1.82	1.92 ± 0.92^*^	3.02 ± 1.78	2.18 ± 1.34^*^	3.46 ± 1.35	3.33 ± 1.51
Fruit	4.12 ± 1.69	5.21 ± 1.67^*^	4.57 ± 1.71	5.25 ± 1.77^*^	4.19 ± 1.48	3.97 ± 1.36

**Notes.**

* *p* < 0.05 Post vs Pre.

Finally, Time × Adiposity Status interaction revealed that the consumption of bread significantly decreased after intervention in normal fat children (4.72 ± 1.47 time/week *vs* 4.08 ± 1.99 time/week, *p* < 0.05), while the consumption of snacks significantly decreased after intervention in normal fat (3.20 ± 1.77 time/week vs 1.94 ± 0.95 time/week) and in obese children (3.07 ± 1.83 time/week vs 2.24 ± 1.43 time/week).

No modifications were found in the consumption of pasta and rice, legumes and milk.

## Discussion

Our study suggested that combined PE and nutritional interventions led to an increase in weekly PA level, a reduction of sedentary time and a qualitative change in the consumption of some specific foods, supporting the efficacy of this type of intervention in primary schools. However, the lack of positive effects on children’s adiposity status confirmed that primary school-based interventions could produce psycho-physical and physiological benefits (Dobbins et al., 2013) but they might have limited effectiveness in the reduction of body weight, BMI and FM% (Verrotti et al., 2014). Specific amounts of PA would be necessary to obtain beneficial changes for skeletal health, aerobic fitness, muscular strength and endurance, and also to reduce adiposity (Strong et al., 2005). Specifically, guidelines for school health PE programs to promote healthy behaviour among children recommended participation in 60 min or more of moderate-to-vigorous enjoyable, developmentally appropriate and varies PA (Strong et al., 2005; US Department of Health and Human Services, 2009). Evidence suggested that school-based PE programs of moderately intense exercise 30–60 min in duration, 3–7 days per week, with a length of training at least three weeks led to a reduction in total body and visceral adiposity in overweight children and adolescents (Strong et al., 2005). Although we respected in both experimental PE interventions the prescribed exercise intensity (MVPA), the overall duration of each lesson (60 min) and the length of training (five months), we did not fully comply these PA guidelines for reasons linked to the scheduling of school activities. Therefore, we supposed that a greater amount of PA in terms of weekly frequency would be necessary to achieve beneficial effects on children’s body composition. In our study, children’s anthropometric values increased after intervention. Growth and maturation processes that occurred during the five months of intervention induced a significant increase of height and also a parallel increase of body weight of children participating in the study. Moreover, the increase of weekly PA level after intervention could have induced a significant increase of lean body mass, and consequently the increase of body weight. Indeed, muscle loading during PA practice increased lean body mass. This increase was due to muscle contraction during physical exercise that acted as a source of mechanical loading and seemed to be beneficial to muscle mass (Nogueira, Weeks & Beck, 2014). Alberga et al. (2013) reported that resistance training increased leg lean mass and leg strength in obese prepubertal youth and positively affected overall PA and health although no changes in fat mass percent were found.

Our results reported that BMI *z*-score did not change after intervention. Even though BMI *z*-score is optimal for assessing adiposity on a single occasion, it is not necessarily the best scale for measuring change in adiposity, as the within-child variability over time depends on the child’s level of adiposity (Cole et al., 2005). Thus, our results emphasized the limitations of BMI *z*-score as a marker of adiposity among children, supporting the use of FM% to track adiposity changes. Nevertheless, we observed an increase of fat mass percentage in children.

According to previous studies (Aburto et al., 2011; Van Grieken et al., 2012), our results revealed that children reduced their sedentary activities and increased the time spent engaged in PA after the intervention period. However, obese children of all three groups appeared to be refractory to change their sedentary behaviour. Previous study showed that obese adolescents fail to conduct an active life because they find limited pleasure in such behaviour (Lindelof, Nielsen & Pedersen, 2013). Therefore, obese children and adolescents need to learn through everyday life a positive attitude toward PA that can lead them to become more active and less sedentary. School-based PE could be a vehicle to provide pleasant and enjoyable experiences for obese children, improving their attitude toward PA, and encouraging physically active behavior.

Finally, our school-based nutritional intervention seems to have induced positive modifications on children’s eating habits. In fact, the consumption of energy-dense foods decreased after intervention, with a contemporary increase of fruit and vegetables consumption, revealing the efficacy of the proposed nutritional intervention to modify eating habits. It seemed that children substituted high energy food and sweet drinks with fruit and vegetables consumption. The presence at school of the European program *“School Fruit Scheme”* stimulated fruit consumption among children. However, our combined PE and nutritional interventions led to a qualitative changes in the eating habits of both experimental groups while no nutritional modifications were found in control group. Our experimental PE interventions contributed to healthy changes in eating habits since ministerial programs of PE for primary school provide experiences aimed to know the relationship between diet, physical exercise and health to assume healthy lifestyle habits (Decree of the President of the Republic of Italy of 16 November 2012, 2013). Although our study showed positive effects of combined PE and nutritional interventions on weekly PA level, sedentary time and eating habits, no improvements of schoolchildren’s body composition were observed. Contrarily to our expectations, no significant reduction was found for weight, BMI *z*-score, and FM% after intervention. The relationship and the compensatory mechanism underlying the interaction between PA and energy intake could affect energy balance (Thivel, Saunders & Chaput, 2013; Thivel & Chaput, 2014), with no positive consequent impacts on anthropometric variables. PA has the potential to modulate appetite control, inducing food consumption modifications (Blundell et al., 2003). PA and eating patterns are closely associated, with a relative strong impact on energy intake leading to an increase in energy intake (Thivel & Chaput, 2014). Moreover, we investigated the weekly food consumption but we did not have indications regarding food portion sizes, which is a key element of energy intake (Livingstone & Pourshahidi, 2014). Therefore, children may probably have increased their food intake since PA might also have increased their hunger and also alter the hedonic response to food (Blundell et al., 2003). A larger-than-appropriate portion size could have increased the fat mass gain in the children participating to the study. It was plausible to assume that the proposed nutritional intervention induced exclusively a positive qualitative changes of nutritional habits of children.

In conclusion, our experimental PE interventions combined with a school nutritional intervention pointed out a better healthful practices in both experimental groups, revealing the efficacy of a multidisciplinary and educational approach.

### Strengths and limitations of this study

The results of this study revealed the effectiveness of an integrated PE and nutritional intervention to improve PA and dietary practices. The strength of the present study included the study design and the large participation of children without drop-out. The intervention was designed to be feasible and not financially demanding for the school system.

However, our research had some limitations. The weekly frequency of PE interventions did not comply guidelines for school health PE programs for reasons linked to the school schedule. Therefore, we sustained the necessity of a congruous involvement of governmental institutions and schools to support children’s healthy habits. Self-reported measures and the use of the OMNI scale for the perceived exertion, could have affected our results. Self-report is a promising methodology for children that is applicable for large studies, but, sometimes, subjects tend to alter the answers (Shepard, 2003). Finally, we investigated the weekly food consumption but we did not have indications regarding food portion sizes, which is a key element of energy intake (Livingstone & Pourshahidi, 2014). Therefore, some changes in children’s energy ingestion could have been occurred, partially explaining some of the discrepancies in the observed results.

## Conclusions

In conclusion, the results of this study revealed the effectiveness of a 5-month combined PE and nutritional intervention to improve schoolchildren’s habits. Since the only school-based intervention was not sufficient to produce significant changes of adiposity status, we sustain the necessity of a congruous involvement of community, schools and families. In particular, the involvement of parents seems to be essential to establish, control, maintain and reinforce patterns of healthful dietary practices, nutritional knowledge and to encourage daily PA to maintain healthy habits.

It would be necessary to conduct a follow-up evaluation to verify the real long-term impact of the school-based interventions on lifestyle of children.

**Tips for physical education at school**

(1) To propose, instead of monotonous and challenging activities, varied, enjoyable, eased, progressive physical activities and in line with the preferences of children to increase motivation and to maximize adherence (e.g., low-intensity aerobic exercise and resistance training could be preferable exercise modalities for overweight and obese children; the usage of music as a distraction can increase exercise tolerance; structured exercise program for health interventions targeting overweight and obese youth).
(2) To propose PE curricula that follow international guidelines for school health PE programs.
(3) To keep students moving during PE classes and during recess break.
(4) To help students become competent in many movement skills to maximize PA adherence.
(5) To use PE to propose an active lifestyle (e.g., to go up stairs; to walk, instead of using transportations; to help the family in the domestic life: housework, gardening).

**Tips for healthy diet at school**

(1) To coordinate healthy eating and PA practices through a school health council and a school health coordinator.
(2) To use a systematic approach to develop, implement, and monitor healthy eating and PA practices.
(3) To encourage consumption of a variety of food from different food groups in the quantities recommended for healthy growth and development.
(4) To place pictures of food portions in school canteens. They might help children to eat right size portions to maintain an optimal body weight.
(5) To encourage fruits and vegetables consumption instead of high energy food (e.g., sweets or sweet drinks) during mid-morning and/or afternoon school breaks substituting drink and snack vending machines with fruit and vegetable centrifuged juice or fresh squeezed orange juice vending machines.

**Key messages**

✓ Combined PE and nutritional interventions were effective to improve children’s healthful dietary practices and to encourage an active lifestyle.
✓ Experimental interventions were more effective in inducing children’s active lifestyle. The practice of new and unusual activities encouraged children’s PA participation in out-of-school settings, reducing sedentary behaviors.
✓ Greater amount of PA in terms of weekly frequency would be necessary to achieve beneficial effects on children’s body composition.
✓ The involvement of schools and parents is essential to establish, control, maintain and reinforce patterns of healthful dietary practices, and to encourage daily PA.

## Supplemental Information

10.7717/peerj.1880/supp-1 Data S1 Raw data file

## Appendix

### Description of an experimental 1 lesson

An experimental 1 lesson consisted of a continuous aerobic circuit training followed by a sub-maximal shuttle run exercise. This lesson focused on the improvement of cardiovascular endurance by varying the types of gaits required (e.g., fast walking, running, skipping) without any specific coordinative request. The experimental 1 lesson required changes in executive modalities and some variations of intensity.

### Description of an experimental 2 lesson

An experimental 2 lesson consisted of sport-unspecific use of basketballs in the context of mini-games. The basketballs were used in unconventional ways with varying game rules (e.g., application of foot-eye coordination techniques with basketballs). This lesson was geared toward the development of both motor control and perceptual-motor adaptation abilities. It focused on the development of psychomotor competences and expertise in movement-based problem solving through functional use of a common tool (e.g., basketball), and considering various tasks that involved decision-making motor tasks and manipulative ball handling skills (e.g., bouncing, throwing, and/or receiving a ball). The experimental 2 lesson combined physical load due to the practice of physical exercises and cognitive load required for movement-based problem solving and decision-making tasks with accurate timing, temporal estimations, temporal production, spatial and temporal adjustments, and spatial and temporal orientation, which are essential cognitive requirements to perform such types of activities. The experimental 2 lesson aimed to develop both motor control abilities and perceptual-motor adaptation abilities, by combining demands on gross-motor and manipulative control abilities and perceptual-motor adaptation abilities (particularly kinesthetic differentiation and response orientation).

## Additional Information and Declarations

### Data Availability

The following information was supplied regarding data availability: Raw data can be found in Data S1.
